# Supplementary material for: Exploring physical activity and patient perceptions in knee osteoarthritis: A mixed-methods study
Source: PLoS One. 2025 Nov 13;20(11):e0336663. doi: 10.1371/journal.pone.0336663 (PMC12614597; doi:10.1371/journal.pone.0336663)
Supplement: Attachment — Submitted filename: Reviewr Comments on -PONE-D-25-20426-.pdf [file pone.0336663.s002.pdf]

PLOS ONE

Exploring Physical Activity and Patient Perceptions in Knee Osteoarthritis: A Mixed-Methods Study

--Manuscript Draft--

**Manuscript Number:**	PONE-D-25-20426
**Article Type:**	Research Article
**Full Title:**	Exploring Physical Activity and Patient Perceptions in Knee Osteoarthritis: A Mixed-Methods Study
**Short Title:**	Knee OA: Activity Levels and Patient Perceptions
**Corresponding Author:**	Moayad Subahi<br>Umm Al-Qura University College of Applied Medical Science<br>Makkah, SAUDI ARABIA
**Keywords:**	knee osteoarthritis, physical activity, physical therapy, patient perceptions, mixed-methods research
**Abstract:**	Knee osteoarthritis is a prevalent condition that affects physical function and quality of life. Physical activity is a foundation of osteoarthritis management; however, patient engagement and their perceptions of treatment options are poorly understood. This study aims to evaluate physical activity levels among individuals with knee osteoarthritis and explore their perceptions, awareness, and experiences with treatment options, particularly physical therapy.<br>A sequential explanatory mixed-methods design was employed. Quantitative data were collected using the International Physical Activity Questionnaire-Short Form from 60 patients to evaluate physical activity levels. Semi-structured interviews with 24 participants explored experiences and perceptions of knee osteoarthritis management. Quantitative data were analyzed descriptively, and qualitative data underwent thematic analysis. Quantitative findings revealed that 90% of participants exhibited low physical activity levels (≤438 METS-min/week), with only 10% achieving moderate or high levels. Qualitative themes highlighted barriers to physical activity, including emotional distress, logistical challenges, and misconceptions about osteoarthritis management. Despite these challenges, support systems and tailored education were identified as facilitators. Patients with knee osteoarthritis demonstrate critically low physical activity levels, influenced by multifaceted barriers. Addressing these barriers through patient-centred interventions, enhanced education, and improved access to physical therapy can promote engagement and improve outcomes
**Order of Authors:**	Moayad Subahi, PhD
	Fahda Alshaikh, PhD
	Eyad Dahlawi, BSc
	Feras zafar
	Tamim Alsulimany
	Nawaf Alnefaie
	Abdulrahman Almalki
**Additional Information:**	
**Question**	**Response**
**Financial Disclosure**<br><br>Enter a financial disclosure statement that describes the sources of funding for the work included in this submission. Review the submission guidelines for detailed requirements. View published research	The author(s) received no specific funding for this work.

Powered by Editorial Manager® and ProduXion Manager® from Aries Systems Corporation

articles from *PLOS ONE* for specific examples.<br><br>This statement is required for submission and **will appear in the published article** if the submission is accepted. Please make sure it is accurate.<br><br>**Funded studies**<br>Enter a statement with the following details:<br>• Initials of the authors who received each award<br>• Grant numbers awarded to each author<br>• The full name of each funder<br>• URL of each funder website<br>• Did the sponsors or funders play any role in the study design, data collection and analysis, decision to publish, or preparation of the manuscript?<br><br>Did you receive funding for this work?	
**Competing Interests**<br><br>Use the instructions below to enter a competing interest statement for this submission. On behalf of all authors, disclose any competing interests that could be perceived to bias this work—acknowledging all financial support and any other relevant financial or non-financial competing interests.<br><br>This statement is required for submission and **will appear in the published article** if the submission is accepted. Please make sure it is accurate and that any funding sources listed in your Funding Information later in the submission form are also declared in your Financial Disclosure statement.<br><br>View published research articles from *PLOS ONE* for specific examples.	The authors have declared that no competing interests exist

**NO authors have competing interests**<br><br>Enter: *The authors have declared that no competing interests exist.*<br><br>**Authors with competing interests**<br><br>Enter competing interest details beginning with this statement:<br><br>*I have read the journal's policy and the authors of this manuscript have the following competing interests: [insert competing interests here]*<br><br>* typeset	
**Ethics Statement**<br><br>Enter an ethics statement for this submission. This statement is required if the study involved:<br><br>• Human participants<br>• Human specimens or tissue<br>• Vertebrate animals or cephalopods<br>• Vertebrate embryos or tissues<br>• Field research<br><br>Write "N/A" if the submission does not require an ethics statement.<br><br>General guidance is provided below. Consult the submission guidelines for detailed instructions. **Make sure that all information entered here is included in the Methods section of the manuscript.**	Ethical approval for the study was obtained from the Local Committee for Biological and Medical Ethics at Umm Al-Qura University (NJDD011023). All participants provided informed consent before participating. Confidentiality and anonymity were ensured through secure data handling and coding systems. Participants could withdraw at any time without impacting their medical care or legal rights, with their data promptly destroyed upon withdrawal

Powered by Editorial Manager® and ProduXion Manager® from Aries Systems Corporation

**Format for specific study types**<br><br>**Human Subject Research (involving human participants and/or tissue)**<br>• Give the name of the institutional review board or ethics committee that approved the study<br>• Include the approval number and/or a statement indicating approval of this research<br>• Indicate the form of consent obtained (written/oral) or the reason that consent was not obtained (e.g. the data were analyzed anonymously)<br><br>**Animal Research (involving vertebrate animals, embryos or tissues)**<br>• Provide the name of the Institutional Animal Care and Use Committee (IACUC) or other relevant ethics board that reviewed the study protocol, and indicate whether they approved this research or granted a formal waiver of ethical approval<br>• Include an approval number if one was obtained<br>• If the study involved *non-human primates*, add *additional details* about animal welfare and steps taken to ameliorate suffering<br>• If anesthesia, euthanasia, or any kind of animal sacrifice is part of the study, include briefly which substances and/or methods were applied<br><br>**Field Research**<br><br>Include the following details if this study involves the collection of plant, animal, or other materials from a natural setting:<br>• Field permit number<br>• Name of the institution or relevant body that granted permission	
**Data Availability**<br><br>Authors are required to make all data underlying the findings described fully available, without restriction, and from the time of publication. PLOS allows rare exceptions to address legal and ethical concerns. See the PLOS Data Policy and FAQ for detailed information.	No - some restrictions will apply

Powered by Editorial Manager® and ProduXion Manager® from Aries Systems Corporation

A Data Availability Statement describing where the data can be found is required at submission. Your answers to this question constitute the Data Availability Statement and **will be published in the article**, if accepted.<br><br>**Important:** Stating 'data available on request from the author' is not sufficient. If your data are only available upon request, select 'No' for the first question and explain your exceptional situation in the text box.<br><br>Do the authors confirm that all data underlying the findings described in their manuscript are fully available without restriction?	
**Describe where the data may be found in full sentences. If you are copying our sample text, replace any instances of XXX with the appropriate details.**<br><br>• If the data are **held or will be held in a public repository**, include URLs, accession numbers or DOIs. If this information will only be available after acceptance, indicate this by ticking the box below. For example: *All XXX files are available from the XXX database (accession number(s) XXX, XXX.).*<br>• If the data are all contained **within the manuscript and/or Supporting Information files**, enter the following: *All relevant data are within the manuscript and its Supporting Information files.*<br>• If neither of these applies but you are able to provide **details of access elsewhere**, with or without limitations, please do so. For example:<br><br>*Data cannot be shared publicly because of [XXX]. Data are available from the XXX Institutional Data Access / Ethics Committee (contact via XXX) for researchers who meet the criteria for access to confidential data.*<br><br>*The data underlying the results presented in the study are available from (include the name of the third party*	Data available on request from the author.

and contact information or URL).<br>• This text is appropriate if the data are owned by a third party and authors do not have permission to share the data.<br><br>* typeset	
Additional data availability information:	

Powered by Editorial Manager® and ProduXion Manager® from Aries Systems Corporation

Manuscript
Click here to access/download;Manuscript;Manuscript.docx

Exploring Physical Activity and Patient Perceptions in Knee Osteoarthritis: A Mixed-Methods Study

Moayad Subahi[1*], Fahda M Alshaikh[1], Eyad M Dahlawi[1], Feras T Zafar[1], Tamim S Alsulimany[1], Nawaf S Alnefaie[1], Abdulrahman H Almalki[1]

[1] Medical Rehabilitation Sciences Department, Faculty of Applied Medical Science, Umm Al-Qura University, Mecca, Saudi Arabia.

[*]**Corresponding author:** Email: mssubahi@uqu.edu.sa

Abstract

Knee osteoarthritis is a prevalent condition that affects physical function and quality of life. Physical activity is a foundation of osteoarthritis management; however, patient engagement and their perceptions of treatment options are poorly understood. This study aims to evaluate physical activity levels among individuals with knee osteoarthritis and explore their perceptions, awareness, and experiences with treatment options, particularly physical therapy. A sequential explanatory mixed-methods design was employed. Quantitative data were collected using the International Physical Activity Questionnaire-Short Form from 60 patients to evaluate physical activity levels. Semi-structured interviews with 24 participants explored experiences and perceptions of knee osteoarthritis management. Quantitative data were analyzed descriptively, and qualitative data underwent thematic analysis. Quantitative findings revealed that 90% of participants exhibited low physical activity levels (≤438 METS-min/week), with only 10% achieving moderate or high levels. Qualitative themes highlighted barriers to physical activity, including emotional distress, logistical challenges, and misconceptions about osteoarthritis management. Despite these challenges, support systems and tailored education were identified as facilitators. Patients with knee osteoarthritis demonstrate critically low physical activity levels, influenced by multifaceted barriers. Addressing these barriers through patient-centred interventions, enhanced education, and improved access to physical therapy can promote engagement and improve outcomes.

Keywords: knee osteoarthritis, physical activity, physical therapy, patient perceptions, mixed-methods research

Introduction

Knee osteoarthritis (OA) is a chronic degenerative joint disease characterized by the progressive breakdown of cartilage and underlying bone within the knee joint [1,2]. It is one of the leading causes of pain and disability worldwide, affecting both middle-aged and elderly populations [3]. Treatment options for knee OA range from conservative non-pharmacological interventions, such as weight management and physical therapy, to pharmacological treatments and surgical procedures [4]. Treatment choice depends on the disease's severity, patient preferences, and associated comorbidities [5].

Physical therapy is essential in managing knee OA, improving joint mobility, strengthening the muscles around the knee, and educating patients on self-management techniques [6]. Several studies have demonstrated that physical therapy can significantly reduce pain and enhance joint function in patients with knee OA [7,8]. Modalities such as therapeutic exercises, manual therapy, heat therapy, electrical stimulation, and assistive devices are key components of effective physical therapy interventions [9,10]. Strengthening exercises, in particular, are essential in these programs, targeting muscles around the knee joint to promote stability, range of motion, and cardiovascular fitness [11].

Physical activity (PA) is also integral to knee OA management, reducing pain, improving joint function, and enhancing quality of life [12,13]. Despite its importance, many individuals with knee OA remain insufficiently active due to barriers such as pain, fear of exacerbating symptoms, and a lack of motivation or accessible exercise facilities [14,15]. Studies emphasize the need for tailored interventions to address these challenges, including educational programs, guided exercise plans, and support from healthcare providers [16,17]. By overcoming these

barriers, patients can engage in regular PA, which is crucial for slowing disease progression and improving long-term outcomes [18].

In Saudi Arabia, limited community awareness about knee OA exacerbates the challenges associated with managing the disease. A cross-sectional study conducted in Jeddah revealed that only 37% of participants correctly identified the mechanism underlying OA, with significant disparities based on educational level [19]. Similarly, a study in Al-Qunfudah reported that most participants had poor (36.1%) or fair (36.8%) understanding of OA, with urban residents and those with higher education demonstrating slightly better awareness [20]. These studies highlight the need for targeted education campaigns, public lectures, and other methods to enhance community knowledge about knee OA. Social media and expert-led websites could serve as effective platforms for disseminating reliable information. However, caution is necessary to prevent misinformation from unreliable sources. Increased awareness through these efforts can empower individuals to adopt preventive measures and seek timely, evidence-based treatments.

Addressing the psychosocial aspects of knee OA is critical for improving patient outcomes. Studies have shown that education and support from family members, physicians, and physical therapists can alleviate anxiety and stress associated with the disease [21]. Patients who receive guidance on weight management, physical activity, and exercise are more likely to adhere to treatment plans and experience improved quality of life [22,23]. Incorporating psychosocial support into multidisciplinary treatment approaches ensures a holistic management strategy that addresses patients' physical and emotional needs [24,25].

Healthcare providers face challenges managing knee OA due to patients' lack of awareness and misconceptions about the disease. Studies maintained that uncertainty among clinicians results from inconsistent treatment strategies and patient distrust [26–28]. This highlights the importance of providing comprehensive education to patients throughout the conservative treatment process. Additionally, fostering trust and clear communication between patients and providers is essential for improving adherence to therapy and achieving better outcomes [29]. The literature reveals significant gaps in awareness and understanding of knee OA, particularly in Saudi Arabia, which hinder effective management and highlight the need for targeted educational efforts. These efforts should aim to empower patients with knowledge, address physical and psychological barriers, and promote evidence-based practices to mitigate the burden of knee OA while paving the way for improved multidisciplinary care.

Materials and Methods

Study Design:

This study used an explanatory sequential mixed methods design to integrate quantitative and qualitative approaches to comprehensively evaluate PA levels and patient experiences in knee OA.

Setting:

The study was conducted in clinical and community settings in Saudi Arabia. Recruitment involved local clinics, hospitals, and community outreach programs to ensure diverse participant representation.

Participants:

Participant recruitment started from 25/Oct/2023 to 25/Feb/2024. Sixty patients with knee OA were recruited for the quantitative component. Inclusion criteria included adults aged 45 years or older with a physician-confirmed diagnosis of knee OA, as per radiographic or clinical evaluation, who could provide informed consent and participate in the assessments. Participants were required to have been under OA management for at least six months to ensure sufficient experience with treatment modalities. For the qualitative component, 24 participants were purposefully selected from the quantitative cohort using maximum variation sampling. Participants were chosen based on differences in age, gender, physical activity levels, and treatment experiences to ensure a broad range of perspectives and enhance the richness of the data. No prior relationships existed between the researcher and participants.

Exclusion criteria for both components included individuals with cognitive impairments, coexisting neurological disorders, or other severe comorbidities that could interfere with participation. Participants who agreed to participate received the participant information sheet and consent form, completed before the data collection began.

Descriptive data of the 24 qualitative participants, including age, gender, BMI category, and number of physiotherapy sessions, were summarized to provide contextual understanding. Each participant was assigned a code (e.g., P01, P02) used when quoting in the results to maintain anonymity while ensuring traceability of responses.

Quantitative Data Collection:

PA levels were assessed using the -Short Form (IPAQ-sf), a validated tool for estimating weekly activity in METS-min/week. Participants were categorized as having low (≤438 METS-min/week), moderate (1100–3500 METS-min/week), or high (>4500 METS-min/week) PA levels [30,31]. Data were collected before face-to-face qualitative interviews or via structured phone interviews.

Qualitative Data Collection:

Semi-structured interviews were conducted with 24 participants in Arabic to explore their experiences, perceptions, and barriers to OA management. An interview guide with open-ended questions was used, developed by the research team based on literature and clinical experience. Interviews were conducted in person or by phone, depending on participant preference, and lasted between 30 to 60 minutes. All interviews were audio-recorded with consent, transcribed verbatim, and translated into English. Transcripts were checked for accuracy and anonymized

before analysis. An interview guide with open-ended questions was used to facilitate discussions in Arabic.

Data Analysis:

Quantitative data were analyzed using descriptive statistics to summarize PA levels. The qualitative data were analyzed using Braun and Clarke's six-step thematic framework [32]. First, transcripts were read and re-read for familiarization. Initial codes were generated inductively by two researchers independently, focusing on semantic and latent content. These codes were then collated into themes, which were reviewed, refined, and discussed among the research team until consensus was reached. NVivo software was used to support data organization.

Themes emerged inductively from the data, reflecting participants' lived experiences. Integrating both data types provided a holistic understanding of the challenges and facilitators in OA management. Reflexivity was maintained throughout the process, with researchers documenting assumptions and reflecting on their influence on data interpretation [33]. Each theme was illustrated with quotes linked to individual participants using pseudonyms (e.g., P01, female, age 55). As a team of rehabilitation scientists and physiotherapists based in Saudi Arabia, we brought expertise in physical therapy and an understanding of the cultural significance of activities like prayer, which shaped our interview guide and interpretation of participants' experiences. To mitigate potential bias toward favouring physical therapy as a treatment modality, we actively sought diverse perspectives during interviews and ensured that thematic analysis remained grounded in participants' own words rather than preconceived assumptions. Interviews were continued until thematic saturation was achieved, as no new themes emerged in the final interviews.

Trustworthiness:

The trustworthiness of the qualitative findings was ensured through targeted strategies[34]. **Credibility** was enhanced by triangulating qualitative themes with quantitative physical activity data from the IPAQ-sf, validating participants' experiences. **Dependability** was achieved by documenting Braun and Clarke's six-step thematic analysis process, providing a clear audit trail. **Confirmability** was supported by having two researchers independently code transcripts and resolve discrepancies, minimizing bias. **Transferability** was addressed through detailed descriptions of the 24 diverse participants and the Saudi Arabian context, enabling applicability assessments. These steps ensured the findings' rigour and authenticity. The qualitative reporting was guided by the Consolidated Criteria for Reporting Qualitative Research (COREQ) checklist to enhance transparency and comprehensiveness.

Ethical Considerations:

Ethical approval for the study was obtained from the Local Committee for Biological and Medical Ethics at Umm Al-Qura University (NJDD011023). All participants provided written informed consent before participating. Participants who could not attend face-to-face interviews, a verbal consent was recorded during the call. Confidentiality and anonymity were ensured through secure data handling and coding systems. Participants could withdraw at any time without impacting their medical care or legal rights, with their data promptly destroyed upon withdrawal.

Results:

Quantitative results

Descriptive statistics:

The study aimed to evaluate physical activity levels among individuals with knee OA and explore their perceptions, awareness, and experiences with treatment options, particularly physical therapy. The quantitative study included 60 participants, with an equal distribution of males (50%) and females (50%). The mean age of the participants was 55.5 years (SD = 6.4, range: 45–65 years). Participants' body mass index (BMI) varied between 19.5 and 47.3, with a mean of 27.8 (SD = 5.8), categorizing the majority as overweight or obese. Regarding educational level, 45% of participants held a bachelor's degree, 20% had a diploma, 16.7% had completed a Master's degree, and 5% had obtained a PhD. Moreover, participants attended an average of 10.8 physiotherapy sessions (SD = 5.7), ranging from 3 to 20 sessions. **Table 1.** presents descriptive statistics of the quantitative study participants.

Table 1. Descriptive statistics of the participants

**Variable**	**Mean (± Standard Deviation)**
**Age (years)**	55.5 (± 6.4)
**Gender**	Male: n=30 (50%), Female: n=30 (50%)
**BMI (kg/m^2)**	27.8 (± 5.8)<br>Normal n=21 (35%)<br>Overweight n=22 (36.6%)<br>Obese n=17 (28.3%)
**Education**	Bachelor's Degree: 17 (45%), Diploma: 12 (20.0%), Master's Degree: 10 (16.7%), High School: 8 (13.3%), PhD: 3 (5.0%)

Physical activity level

The findings indicated that PA levels in the knee OA population were significantly low. Among the 60 participants, the median total METS-min/week was 66, with 90% of the cohort having low activity levels (≤438 METS-min/week). Only six participants demonstrated moderate (1100–3500 METS-min/week) or high (>4500 METS-min/week) levels of PA. These results highlight the predominance of sedentary behaviour among individuals with OA, highlighting a critical gap in adherence to PA guidelines. **Table 2.** presents the results of physical activity.

Table 2. Physical activity level results

Physical activity level	Number of participants (%percentage)
**Low (≤438 METS-min/week)**	54 (90%)
**Moderate (1100–3500 METS-min/week)**	4 (6.6%)
**High (>4500 METS-min/week)**	2 (3.3%)

Qualitative results

The qualitative study resulted in six themes developed through the thematic analysis: The beginning of the patient's journey, Treatment, Providing care for individuals with knee OA, The patient's therapeutic culture, Patient environment, Challenges and Barriers. **Table 3.** Themes, subthemes, and exemplary quotes from the patients interview. The qualitative component highlighted the underlying reasons for these low activity levels, revealing multifaceted barriers. Emotional distress, often associated with chronic pain and reduced mobility, was frequently cited as a deterrent to maintaining regular activity. Logistical challenges, such as difficulty accessing physical therapy services and a lack of transportation,

further compounded these barriers. Participants also expressed limited awareness about the benefits of exercise and common misconceptions about OA management, leading to a reliance on passive treatments and avoidance of physical activity.

Table 3. Themes, subthemes, and exemplary quotes from the patients interview

**Theme 1: The Beginning of the Patient's Journey**	
**The Onset of the Disease**	"I could not bend my knee, especially during prayer; I could not sit properly in prayer."
**Decision-Making**	"Honestly, I personally prefer physical therapy over surgery. I'd rather avoid surgery."
**Theme 2: Treatment**	
**The Patient's Preference for Therapeutic Solutions**	"Honestly, I personally prefer physical therapy over surgery. I'd rather avoid surgery and any surgical procedures. Surgery should be the last option, only if there's no other solution I will do it. but I prefer to do physical therapy even if it takes longer".
**Interest in Returning to Life**	"Thank God, I continue to take one ibuprofen pill a day and one vitamin B12 pill."

**Exercise**	"My experience with the exercises given to me by the physiotherapist at the hospital has been very satisfying. The pain has significantly decreased and improved a lot."
**Theme 3: Providing Care for Individuals with OA**	
**Physical Therapy: Perceptions and Reality**	"The doctor said the surgery was 100% successful, but 70% of the recovery depends on physical therapy."
**Access and Quality of Care**	"The doctor would call me online and conduct a virtual physical therapy session."
**From Goals to Gains: Patient's Progress and Outcomes**	"After undergoing physical therapy, I felt relieved, and I was able to walk on my leg again."
**Theme 4: The Patient's Therapeutic Culture**	
**Curiosity and Awareness: Understanding Knee OA**	"Is this surgery beneficial or not? Tell me, have many people been able to walk perfectly?"
**Options and Experiences: Perspectives on Care**	"They just give brief instructions and don't even follow up on some of the exercises."
**Theme 5: Patient Environment**	
**Community Support: A Pillar of Healing**	"I don't have anyone. Honestly, my friends, my husband, and my children help me."
**Myths and Misconceptions: Navigating Patient Advice**	"How does physical therapy work, especially for knee OA? And how can cracking sound go away?"

Theme 6: Challenges and Barriers	
**Other Diseases and Injuries**	"I had an accident 25 years ago that caused complications in my knee."
**Obstacles and Emotions**	"Living with knee OA significantly affected patient's mental and emotional health."

Despite these barriers, the qualitative analysis also uncovered facilitators that encouraged engagement in PA. Support from family members and healthcare providers played a significant role in motivating participants to adhere to treatment plans and exercise routines. Additionally, participants who received tailored education about OA and the role of exercise reported greater confidence in their ability to manage symptoms through activity. Those with access to consistent PT services noted significant improvements in pain, mobility, and overall quality of life, emphasizing the importance of structured, guided interventions.

Integrating the quantitative and qualitative findings, this study highlights a critical need to address the barriers to physical activity in OA populations. While quantitative data highlight the severity of inactivity, qualitative insights provide a deeper understanding of patients' emotional, logistical, and educational challenges. Tailored interventions prioritizing patient education, emotional support, and accessible physical therapy services are essential to improving PA levels and overall disease management.

Theme 1: The beginning of the patient's journey

The onset of knee osteoarthritis marked a significant disruption in participants' lives, as chronic knee pain began to impede daily activities imbued with personal and cultural significance. One participant shared, "I could not bend my knee, especially during prayer; I could not sit properly

in prayer," highlighting how the condition challenged their ability to engage in religious practices central to life in Saudi Arabia. This physical limitation often triggered a cascade of emotional distress and reduced quality of life, prompting participants to seek medical advice. Consultations with orthopaedic specialists confirmed the diagnosis and introduced treatment options, with physical therapy frequently recommended as a first step. When choosing a path forward, participants leaned toward conservative approaches over surgery. As one explained, "Honestly, I personally prefer physical therapy over surgery. I'd rather avoid surgery," reflecting a common fear of invasive procedures and a hope that physical therapy could restore function less disruptively. These early experiences reveal how physical symptoms, cultural priorities, and treatment preferences shaped participants' initial steps in managing knee OA, setting the stage for their engagement or lack of physical activity.

Theme 2: Treatment

Participants' approaches to treatment were shaped by a complex mix of preferences, practical considerations, and aspirations to reclaim their pre-OA lives. Many expressed a strong inclination toward physical therapy over surgical interventions, driven by both psychological comfort and perceived effectiveness. One participant expressed this clearly: "Honestly, I personally prefer physical therapy over surgery. I'd rather avoid surgery and any surgical procedures. Surgery should be the last option, only if there's no other solution I will do it. But I prefer to do physical therapy even if it takes longer." This quote highlights a deliberate choice to prioritize less invasive methods despite the time commitment, reflecting a broader reluctance to embrace surgery unless necessary. For those who adhered to treatment plans often combining medication and physical therapy—the rewards were evident. Another participant noted, "Thank God, I continue to take one ibuprofen pill a day and one vitamin B12 pill," suggesting that a manageable regimen supported their persistence.

Exercise, a cornerstone of physical therapy, elicited varied responses. For some, it was transformative: "My experience with the exercises given to me by the physiotherapist at the hospital has been very satisfying. The pain has significantly decreased and improved a lot." This positive outcome highlights how tailored therapeutic routines can bridge the gap between sedentary behaviour (noted in 90% of participants with ≤438 METS-min/week) and improved function. However, logistical barriers tempered the enthusiasm of others, who found attending sessions inconvenient and yearned for at-home alternatives. This tension between the benefits of exercise and the challenges of accessing it points to a critical need for flexible, patient-centred solutions to enhance adherence and activity levels.

Theme 3: Providing care for individuals with knee OA.

Participants' experiences with care delivery, particularly physical therapy, revealed a spectrum of perceptions that evolved with exposure to treatment. Initially, some believed in disbelief or confusion about its efficacy, but tangible improvements often shifted their views. One participant reflected on this journey: "The doctor said the surgery was 100% successful, but 70% of the recovery depends on physical therapy." This insight, gained post-surgery, highlights the pivotal role of physical therapy in achieving functional outcomes, reinforcing its value beyond initial expectations. Access to quality care further shaped experiences. Innovative solutions like virtual sessions were a boon for some: "The doctor would call me online and conduct a virtual physical therapy session", offering flexibility amid logistical challenges like transportation or long wait times. However, inconsistent communication and appointment delays frustrated others, highlighting disparities in care delivery.

When physical therapy aligned with participants' goals, the gains were palpable. "After undergoing physical therapy, I felt relieved, and I was able to walk on my leg again," one participant shared, illustrating how consistent engagement could restore mobility and alleviate pain. However, missed sessions or neglected home exercises disrupted progress, emphasizing that the benefits of care hinge on sustained participation. These findings suggest that while physical therapy holds promise for improving low activity levels, its success depends on overcoming barriers to access and fostering patient-provider collaboration.

Theme 4: The patient's therapeutic culture.

Participants' therapeutic culture of knowledge, curiosity, and attitudes toward knee OA management revealed gaps and glimmers of awareness that influenced their treatment journeys. Many sought clarifications on options, asking questions like, "Is this surgery beneficial? Tell me, have many people been able to walk perfectly?" This curiosity reflects an active desire to understand their condition, yet it often coexisted with limited baseline knowledge about OA and its management. Physical therapy, however, emerged as a point of recognition for some, who valued its role in strengthening muscles and stabilizing joints, even if their understanding was incomplete.

Experiences with healthcare systems further shaped perspectives. Dissatisfaction surfaced when care felt cursory: "They just give brief instructions and don't even follow up on some of the exercises," one participant lamented, pointing to a perceived lack of personalization in public settings. In contrast, those who received detailed explanations or accessed private care expressed greater satisfaction, suggesting that quality of interaction influences trust and engagement. Despite these challenges, hope persisted: "I believe consistent physical therapy can improve my quality of life," another noted, indicating that education and positive care

experiences could empower patients to embrace evidence-based practices over time. This theme highlights a critical need for targeted education to bridge knowledge gaps and align therapeutic culture with effective OA management.

Theme 5: Patient environment

The patient's environment of family, friends, and community served as both a pillar of support and a source of complexity in their recovery journey. Emotional and practical encouragement from loved ones was invaluable: "I don't have anyone. Honestly, my friends, my husband, and my children help me," one participant shared, emphasizing how social networks mitigated isolation and bolstered adherence to treatment plans. Community interactions, however, introduced a mix of helpful and misguided advice. Traditional remedies, passed down through generations, held appeal for some, yet often lacked evidence. Another participant questioned prevailing myths: "How does physical therapy work, especially for knee OA? And how can cracking sound go away?" This reflects a broader struggle to navigate well-intentioned but unsupported suggestions, such as dietary fixes or unproven treatments, which sometimes affect evidence-based options.

These environmental dynamics illuminate why many participants remained sedentary. Community misconceptions reinforced avoidance of activity, while supportive networks offered a counterbalance that encouraged persistence. Addressing this duality requires education that extends beyond the individual to their social circles, ensuring that support aligns with clinical recommendations and fosters greater physical activity engagement.

Theme 6: Challenges and Barriers

Living with knee OA presented multifaceted challenges that compounded physical limitations with emotional and logistical hurdles. Coexisting conditions amplified difficulties: "I had an accident 25 years ago that caused complications in my knee," one participant explained, illustrating how past injuries or comorbidities complicated recovery and deterred activity. External disruptions, like the COVID-19 pandemic, further derailed care, forcing delays or abandonment of therapy sessions and exacerbating sedentary tendencies (reflected in the 90% low activity rate).

The emotional toll was equally profound. "Living with knee OA significantly affected my mental and emotional health," another participant confessed, capturing the frustration, anxiety, and isolation that stemmed from restricted mobility. This psychological burden often reinforced a cycle of inactivity, as pain and fear of worsening symptoms deterred exercise. These barriers highlight the need for holistic interventions integrating emotional support, tailored education, and accessible care to break the sedentary pattern and improve outcomes. Participants' narratives reveal that overcoming these challenges is not just a physical attempt but a profoundly personal one, requiring a biopsychosocial approach to OA management.

Integration of quantitative and qualitative results

The qualitative themes provide a nuanced explanation for the quantitative finding that 90% of participants exhibited low physical activity levels (≤438 METS-min/week). Emotional distress and logistical barriers (Theme 6) deterred engagement, while limited awareness and misconceptions (Themes 4 and 5) perpetuated sedentary behaviour. Conversely, support systems (Theme 5) and effective physical therapy experiences (Themes 2 and 3) emerged as facilitators, suggesting pathways to bridge the activity gap identified in the IPAQ-sf data.

Together, these findings highlight the interplay of personal, social, and structural factors in shaping OA management.

Discussion:

This mixed-methods study comprehensively explains PA levels and patient experiences in knee OA management. The findings emphasize the substantial gap in adherence to physical activity guidelines, with 90% of participants classified as having low activity levels. This highlights a critical area for intervention in OA care. The barriers identified in the qualitative analysis align with existing literature, emphasizing the role of emotional distress, logistical challenges, and limited awareness in hindering physical activity. Participants frequently reported that chronic pain, reduced mobility, and misconceptions about OA management contributed to their sedentary behaviour. These findings reinforce the importance of addressing psychological and educational aspects alongside physical symptoms to improve patient outcomes.

Notably, the study highlights facilitators that can enhance physical activity engagement. Support from family members and healthcare providers emerged as a significant motivator, suggesting that a collaborative approach involving the patient's social network could yield positive results. Tailored education about the benefits of physical activity and accessible physical therapy services were also identified as key enablers of improved activity levels. These findings align with the biopsychosocial model of care, which advocates for a holistic approach to managing chronic conditions like OA.

The integration of quantitative and qualitative findings provides robust evidence for the development of targeted interventions. For instance, implementing community-based education programs to address misconceptions and increase awareness about OA management could empower patients to take an active role in their care. Similarly, expanding access to physical therapy services, particularly in underserved areas, could help overcome logistical barriers and improve adherence to exercise regimens.

Future research should focus on evaluating the effectiveness of tailored, multifaceted interventions that address the unique barriers and facilitators identified in this study. Longitudinal studies examining the impact of such interventions on physical activity levels, pain management, and quality of life in OA patients would further strengthen the evidence base.

Limitations:

This study has several limitations that should be acknowledged. First, the sample size for the quantitative and qualitative components was relatively small, which may limit the generalizability of the findings. The study was conducted in a single geographic region, and cultural factors specific to Saudi Arabia may influence the experiences and perceptions of participants. Second, the study's cross-sectional design does not allow for causal inferences regarding the relationship between identified barriers and PA levels. Longitudinal studies are needed to explore these dynamics over time. Finally, the reliance on self-reported measures for PA levels, such as the IPAQ-sf, may introduce recall bias, potentially affecting the accuracy of the results.

Conclusion:

Integrating PA-level assessment with qualitative insights offers a robust understanding of the challenges and facilitators in OA management. Addressing barriers through tailored interventions that include education, emotional support, and accessible physical therapy services can significantly improve adherence to physical activity and overall quality of life in OA patients. This study highlights the need for patient-centred approaches that consider the biopsychosocial dimensions of OA care.

Conflicts of Interest and Source of Funding: This research received no external funding, and the authors declare no conflicts of interest.

Data Availability Statement: Data available on request from the author.

Reference list:

[1] Osteoarthritis Research Society International. Osteoarthritis: A Serious Disease, Submitted to the U. S. Food and Drug Administration. Oarsi 2016.

[2] Kolasinski SL, Neogi T, Hochberg MC, Oatis C, Guyatt G, Block J, et al. American College of Rheumatology/Arthritis Foundation Guideline for the Management of Osteoarthritis of the Hand, Hip, and Knee. Arthritis Rheumatol 2020;0:1–14. https://doi.org/10.1002/art.41142.

[3] Driban JB, Hootman JM, Sitler MR, Harris KP, Cattano NM. Is Participation in Certain Sports Associated With Knee Osteoarthritis? A Systematic Review. J Athl Train 2017;52:497–506. https://doi.org/10.4085/1062-6050-50.2.08.

[4] Osteoarthritis Research Society International. definition of osteoarthritis 2019. https://www.oarsi.org/research/standardization-osteoarthritis-definitions (accessed December 6, 2019).

[5] Wang QF, Ma YF, Yin YS. [A new understanding of the diagnosis and treatment of knee osteoarthritis]. Zhongguo Gu Shang 2016;29:779–81. https://doi.org/10.3969/j.issn.1003-0034.2016.09.001.

[6] Rogers J, Shepstone L, Dieppe P. Is Osteoarthritis a Systemic Disorder of Bone? Arthritis Rheum 2004. https://doi.org/10.1002/art.20136.

[7] Yu SP, Hunter DJ. Managing osteoarthritis. Aust Prescr 2015. https://doi.org/10.1111/1751-486X.12178.

[8] Brosseau L, Taki J, Desjardins B, Thevenot O, Fransen M, Wells GA, et al. The Ottawa panel clinical practice guidelines for the management of knee osteoarthritis. Part three: Aerobic exercise programs ⁎. Clin Rehabil 2017;31:612–24. https://doi.org/10.1177/0269215517691085.

[9] Ytterberg SR, Mahowald ML, Krug HE. Exercise for arthritis. Baillieres Clin Rheumatol 1994. https://doi.org/10.1016/S0950-3579(05)80230-4.

[10] Juhl C, Christensen R, Roos EM, Zhang W, Lund H. Impact of exercise type and dose on pain and disability in knee osteoarthritis: A systematic review and meta-regression analysis of randomized controlled trials. Arthritis and Rheumatology 2014. https://doi.org/10.1002/art.38290.

[11] Fransen M, McConnell S, Harmer AR, Van Der Esch M, Simic M, Bennell KL. Exercise for osteoarthritis of the knee: A Cochrane systematic review. Br J Sports Med 2015. https://doi.org/10.1136/bjsports-2015-095424.

[12] Eckstrom E, Neukam S, Kalin L, Wright J. Physical Activity and Healthy Aging. Clin Geriatr Med 2020;36:671–83. https://doi.org/10.1016/j.cger.2020.06.009.

[13] Willett M, Duda J, Gautrey C, Fenton S, Greig C, Rushton A. Effectiveness of behavioural change techniques in physiotherapy interventions to promote physical activity adherence in patients with hip and knee osteoarthritis: A systematic review protocol. BMJ Open 2017;7:1–9. https://doi.org/10.1136/bmjopen-2017-015833.

[14] Qin J, Barbour KE, Nevitt MC, Helmick CG, Hootman JM, Murphy LB, et al. Objectively Measured Physical Activity and Risk of Knee Osteoarthritis. Med Sci Sports Exerc 2017:1. https://doi.org/10.1249/MSS.0000000000001433.

[15] Coste N, Guiguet-Auclair C, Gerbaud L, Pereira B, Berland P, Gay C, et al. Perceived barriers to and facilitators of physical activity in people with knee osteoarthritis: Development of the Evaluation of the Perception of Physical Activity questionnaire. Ann Phys Rehabil Med 2020. https://doi.org/10.1016/j.rehab.2019.07.009.

[16] Stevenson JD, Roach R. The benefits and barriers to physical activity and lifestyle interventions for osteoarthritis affecting the adult knee. J Orthop Surg Res 2012. https://doi.org/10.1186/1749-799X-7-15.

[17] Kanavaki AM, Rushton A, Efstathiou N, Alrushud A, Klocke R, Abhishek A, et al. Barriers and facilitators of physical activity in knee and hip osteoarthritis: a systematic review of qualitative evidence. BMJ Open 2017. https://doi.org/10.1136/bmjopen-2017-017042.

[18] Gay C, Chabaud A, Guilley E, Coudeyre E. Educating patients about the benefits of physical activity and exercise for their hip and knee osteoarthritis. Systematic literature review. Ann Phys Rehabil Med 2016. https://doi.org/10.1016/j.rehab.2016.02.005.

[19] Alyami AH, Alswat MM, Omer IA, Ahmed MEH, Alshammari SH, Alsaggaf KW, et al. General population knowledge about osteoarthritis and its related risk factors in Jeddah Saudi Arabia. Saudi Med J 2020;41:516–23. https://doi.org/10.15537/smj.2020.5.25061.

[20] Alkalash SH, Alsyed AA, Alrashdi AA, Alqarni AS, Alessa MA, Alzubaidi HH, et al. Public Knowledge of Osteoarthritis in Al-Qunfudah Governorate, Saudi Arabia. Cureus 2023;15:e34892. https://doi.org/10.7759/cureus.34892.

[21] Juby AG, Skeith K, Davis P. Patients' awareness, utilization, and satisfaction with treatment modalities for the management of their osteoarthritis. Clin Rheumatol 2005. https://doi.org/10.1007/s10067-005-1102-0.

[22] Nicolson PJA, Hinman RS, French SD, Lonsdale C, Bennell KL. Improving Adherence to Exercise: Do People With Knee Osteoarthritis and Physical Therapists Agree on the Behavioral Approaches Likely to Succeed? Arthritis Care Res (Hoboken) 2018;70:388–97. https://doi.org/10.1002/acr.23297.

[23] Willett M, Duda J, Fenton S, Gautrey C, Greig C, Rushton A. Effectiveness of behaviour change techniques in physiotherapy interventions to promote physical activity adherence in lower limb osteoarthritis patients: A systematic review. PLoS One 2019;14:1–23. https://doi.org/10.1371/journal.pone.0219482.

[24] Hunter DJ, Schofield D, Callander E. The individual and socioeconomic impact of osteoarthritis. Nat Rev Rheumatol 2014. https://doi.org/10.1038/nrrheum.2014.44.

[25] Kauppila AM, Kyllönen E, Mikkonen P, Ohtonen P, Laine V, Siira P, et al. Disability in end-stage knee osteoarthritis. Disabil Rehabil 2009. https://doi.org/10.1080/09638280801976159.

[26] Subahi M. An evaluation of physical fitness and physical activity to support therapeutic exercise prescription for individuals with knee osteoarthritis in Saudi Arabia: a mixed-methods study. Cardiff University, 2021.

[27] Albert FA, Crowe MJ, Malau-Aduli AEO, Malau-Aduli BS. Physical activity promotion: A systematic review of the perceptions of healthcare professionals. vol. 17. 2020. https://doi.org/10.3390/ijerph17124358.

[28] Al-Abbad HM, Al-Haidary HM. The perception of physical therapy leaders in Saudi Arabia regarding physical therapy scope of practice in primary health care. J Phys Ther Sci 2016. https://doi.org/10.1589/jpts.28.112.

[29] Selten EM, Vriezekolk JE, Geenen R, van der Laan WH, van der Meulen-Dilling RG, Nijhof MW, et al. Reasons for Treatment Choices in Knee and Hip Osteoarthritis: A Qualitative Study. Arthritis Care Res (Hoboken) 2016;68:1260–7. https://doi.org/10.1002/acr.22841.

[30] Ács P, Betlehem J, Oláh A, Bergier J, Melczer C, Prémusz V, et al. Measurement of public health benefits of physical activity: Validity and reliability study of the international physical activity questionnaire in Hungary. BMC Public Health 2020;20:1–10. https://doi.org/10.1186/s12889-020-08508-9.

[31] Mehta SP, Jarvis A, Standifer D, Warnimont C. International physical activity questionnaire. Crit Rev Phys Rehabil Med 2018;30:125–7. https://doi.org/10.1615/CritRevPhysRehabilMed.2018026180.

[32] Clarke V, Braun V. Thematic analysis. In Encyclopedia of critical psychology. Encyclopedia of critical psychology, 2014.

[33] Williamson K, Given LM, Scifleet P. Qualitative data analysis. Research Methods: Information, Systems, and Contexts: Second Edition, 2018. https://doi.org/10.1016/B978-0-08-102220-7.00019-4.

[34] Connelly LM. Trustworthiness in qualitative research. MEDSURG Nursing 2016;25:435–6.
