# Supplementary material for: Exploring physical activity and patient perceptions in knee osteoarthritis: A mixed-methods study
Source: PLoS One. 2025 Nov 13;20(11):e0336663. doi: 10.1371/journal.pone.0336663 (PMC12614597; doi:10.1371/journal.pone.0336663)
Supplement: Attachment — Submitted filename: PONE-D-25-20426.docx [file pone.0336663.s001.docx]

Manuscript Title: Exploring Physical Activity and Patient Perceptions in Knee Osteoarthritis: A Mixed-Methods Study

Manuscript ID: PONE-D-25-20426

An excellent sequential explanatory mixed-methods study of physical activity levels and patient opinions of knee osteoarthritis (OA) management in Saudi Arabia is presented in this publication. Because it focuses on a Middle Eastern population, the study is methodologically sound, ethically appropriate, and contextually meaningful. Both quantitative and qualitative data are well integrated. Small changes are required to improve data openness, clarify methods, and focus the conversation.

**Introduction:**

1. Novelty could be more clearly distinguished from previous mixed-methods studies in OA.

**Methodology:**

1. The cutoff for “low activity” (≤438 MET-min/week) using IPAQ-SF is non-standard. Please clarify and cite validation studies for this threshold.
2. A **sample size justification** for the quantitative phase (n=60) is missing.

**Results and Data Presentation**

1. Table formatting (Table 1, Table 2) should be cleaned for clarity and consistency.
2. Avoid duplicating large blocks of quotes in both Table 3 and the Results section – summarize selectively.

**Discussion:**

1. Potential gender-based insights, given equal male/female participation, are not discussed.

**Data Availability**

Current statement ("data available on request") does not meet PLOS ONE’s data availability policy. Authors must deposit data in a public repository or provide detailed justification for restrictions.

**Reference:**

Ensure full and consistent formatting (some DOIs and titles are not uniform).
