# Supplementary material for: Exploring physical activity and patient perceptions in knee osteoarthritis: A mixed-methods study
Source: PLoS One. 2025 Nov 13;20(11):e0336663. doi: 10.1371/journal.pone.0336663 (PMC12614597; doi:10.1371/journal.pone.0336663)
Supplement: Attachment — Submitted filename: Response to Reviewers.docx [file pone.0336663.s004.docx]

# Response to Reviewers

We sincerely thank the Academic Editor and Reviewers for their constructive feedback. We have carefully revised our manuscript in response to all comments. Below we provide a detailed, point-by-point response, with changes highlighted in the revised manuscript and page/line numbers indicated.

## Reviewer #1

| **Reviewer Comment** | **Author Response** | **Manuscript Location (Revised)** |
| --- | --- | --- |
| Lack of theoretical framework, research paradigm, and positionality. | Added explicit statement of **pragmatic paradigm**, reflexivity, and researcher backgrounds; COREQ reporting confirmed. | Page 6, Lines 1–10; Page 8–9, Lines 5–40 |
| No sample size justification (quantitative and qualitative). | Explained no a priori power calculation; rationale for 60 participants (descriptive precision); 24 interviews achieved saturation. | Page 6, Lines 30–40; Page 9, Line 35 |
| Limited integration of findings. | Strengthened integration by linking low PA (quant) with barriers/facilitators (qual). | Page 15, Lines 5–25 |
| Quantitative analysis restricted to descriptive. | Added exploratory subgroup analyses (gender, BMI, education); none significant. | Page 10, Lines 5–20 |
| Qualitative analysis transparency limited. | Expanded methods: Braun & Clarke’s six steps, NVivo 12, peer debriefing, reflexivity. | Page 8–9, Lines 5–40 |
| Recommendation: Major Revision. | All above points addressed to improve rigor and transparency. | Throughout methods & results |
| Reviewer Comment | Author Response | Manuscript Location (Revised) |
| Inconsistent capitalization in themes. | Standardized all theme/subtheme titles to Title Case. | Page 12–14, Table 3 |
| Misses recent global guidelines (NICE, EULAR 2023). | Added NICE (NG226, 2022), OARSI, and EULAR 2023 in Introduction/Discussion. | Page 3, Lines 20–30; Page 17, Lines 5–20 |

## Reviewer 2

| **Reviewer Comment** | **Author Response** | **Manuscript Location (Revised)** |
| --- | --- | --- |
| Inconsistent capitalization in themes. | Standardized all theme/subtheme titles to Title Case. | Page 12–14, Table 3 |
| Misses recent global guidelines (NICE, EULAR 2023). | Added NICE (NG226, 2022), OARSI, and EULAR 2023 in Introduction/Discussion. | Page 3, Lines 20–30; Page 17, Lines 5–20 |
| Sample size justification missing. | Added rationale (exploratory design). | Page 6, Lines 30–40 |
| Clearer alignment between qual themes and quant data. | Integration section strengthened to link IPAQ results with themes. | Page 15, Lines 5–25 |

## Reviewer #3

| **Reviewer Comment** | **Author Response** | **Manuscript Location (Revised)** |
| --- | --- | --- |
| Purpose of mixed methods unclear. | Clarified rationale: sequential explanatory design. | Page 5, Lines 20–30 |
| Was it descriptive only? | Confirmed exploratory descriptive nature. | Page 6, Lines 25–30 |
| IPAQ-SF validation? | Added references for reliability/validity in KOA and Arabic populations. | Page 7, Lines 5–15 |
| Sample size justification. | Added rationale. | Page 6, Lines 30–40; Page 9, Line 35 |
| Data repeated in text and tables. | Shortened text; details remain in tables. | Page 10–11 |
| Participant characteristics table missing. | Added **Table 1**. | Page 10, Table 1 |
| Quotes not linked to participants. | Added pseudonyms (e.g., P01, female, 55). | Page 12–14, Table 3 |
| Discussion needs reinforcement. | Expanded comparisons with global studies. | Page 16–17 |

## Reviewer #4

| **Reviewer Comment** | **Author Response** | **Manuscript Location (Revised)** |
| --- | --- | --- |
| Abstract lacks demographics and recruitment strategy. | Added age, sex, BMI, and recruitment (clinics + community). | Page 2, Lines 20–30 |
| Intro missing prevalence, etiology, psychosocial factors. | Added Saudi prevalence, etiological contributors, psychosocial burden. | Page 3, Lines 5–20 |
| Poor phrasing of education–awareness link. | Rewritten to show graded relationship. | Page 4, Lines 15–25 |
| Cite recent studies. | Added 2023–2024 references. | Page 4, Lines 20–25 |
| Methods: unclear participant selection/flow. | Reorganized section for clarity. | Page 6–7 |
| IPAQ-SF details missing. | Added full name, units, formula, and categories. | Page 7, Lines 5–20 |
| Qualitative details missing (translation, interviewer, guide source). | Added translation process, interviewer role, bias mitigation, and guide source. | Page 8, Lines 10–20 |
| NVivo version missing. | Added NVivo 12. | Page 8, Line 25 |
| Trustworthiness references missing. | Added COREQ, IPAQ-SF references. | Page 9, Lines 10–20 |
| Results need min–max values. | Added ranges for age, BMI, sessions. | Page 10, Lines 5–15 |
| Report n for moderate/high PA. | Added counts. | Page 11, Lines 15–20 |
| Add references for qualitative affirmations. | Added citations. | Page 13, Lines 20–30 |
| Integration needs Creswell & Plano Clark. | Added reference. | Page 15, Line 20 |
| Discussion lacks references. | Added global/regional comparisons. | Page 16–18 |
| Use KOA consistently. | Corrected throughout. | Whole manuscript |
| English requires improvement. | Revised for clarity. | Whole manuscript |

## Reviewer #5

| **Reviewer Comment** | **Author Response** | **Manuscript Location (Revised)** |
| --- | --- | --- |
| Novelty not well distinguished. | Stated novelty: first Saudi sequential explanatory study integrating IPAQ-SF with perceptions. | Page 4–5 |
| IPAQ-SF cut-off non-standard. | Corrected cut-offs and updated results. | Page 7, Lines 5–20; Results |
| Sample size justification missing. | Added rationale. | Page 6, Lines 30–40 |
| Tables inconsistent. | Reformatted Tables 1 & 2. | Page 10–11 |
| Quotes duplicated in text and table. | Shortened text, left full quotes in Table 3. | Page 12–14 |
| Gender-based insights absent. | Added gender paragraph. | Page 17, Lines 10–20 |
| Data availability inadequate. | Revised to institutional contact. | Page 21 |
| References inconsistent. | Revised all references. | Reference list |

## Reviewer #6

| **Reviewer Comment** | **Author Response** | **Manuscript Location (Revised)** |
| --- | --- | --- |
| Self-report bias in IPAQ-SF. | Acknowledged in limitations; suggested accelerometers. | Page 19, Lines 5–15 |
| Cross-sectional design prevents causality. | Added as limitation; suggested longitudinal follow-up. | Page 19, Lines 15–25 |
| Thematic analysis depth limited. | Expanded subthemes, added more quotes. | Page 12–14 |
| Data availability insufficient. | Revised to institutional contact. | Page 21 |
| Discussion alignment with literature weak. | Reorganized to compare findings with prior studies. | Page 16–18 |
| Language clarity. | Revised grammar and style. | Whole manuscript |

## Reviewer #7

| **Reviewer Comment** | **Author Response** | **Manuscript Location (Revised)** |
| --- | --- | --- |
| Conclusions overstated. | Softened: “may support engagement” instead of “significantly improve QoL.” | Page 18, Lines 20–30 |
| “Critically low” unclear. | Replaced with standard “low PA (<600 MET-min/week).” | Page 11; Page 18 |
| Terminology suggests intervention. | Clarified study is cross-sectional and exploratory. | Page 5, Lines 20–30; Abstract |
| Demographic factors not discussed. | Added exploratory results and discussion. | Page 10; Page 17 |
| Generalizations too broad. | Tempered; emphasized Saudi-specific context. | Page 18–19 |
| Methods – saturation not explained. | Added: saturation reached when no new codes emerged. | Page 9, Lines 30–35 |
| Perceptions/awareness only qualitatively assessed. | Clarified scope in abstract and methods. | Page 2, Lines 20–30; Page 5, Lines 15–25 |

## Academic Editor

| Reviewer Comment | Author Response | Manuscript Location (Revised) |
| --- | --- | --- |
| Data Availability Contact | Revised to provide institutional contact (Umm Al-Qura University Ethics Committee, generic contact email). | Page 21 |

**Required files prepared:**

- *Response to Reviewers* (this document).
- *Revised Manuscript with Track Changes*.
- *Clean Manuscript (no track changes)*.
